# Does Prophylactic Foraminotomy Reduce Postoperative C5 Palsy in Posterior Reconstruction Surgery Using Cervical Pedicle Screw?

**DOI:** 10.7759/cureus.112131

**Published:** 2026-07-06

**Authors:** Terumasa Ikeda, Hiroshi Miyamoto

**Affiliations:** 1 Orthopedic Surgery, Kindai University Hospital, Sakai, JPN; 2 Orthopedic Surgery, Kobe Rosai Hospital, Kobe, JPN

**Keywords:** cervical correction surgery, cervical pedicular screw, cervical spine kyphosis, postoperative c5 palsy, prophylactic foraminotomy

## Abstract

Background

Postoperative C5 nerve palsy is a well-recognized complication following posterior cervical decompression and reconstruction; however, its precise mechanism remains unclear. Although prophylactic C4-5 foraminotomy has been proposed as a preventive strategy, its effectiveness remains controversial.

Methodology

A total of 37 patients who underwent cervical pedicle screw (CPS)-based corrective surgery (21 males and 16 females; mean age, 71 years) were included. Prophylactic foraminotomy at C4-5 was performed in patients with preoperative foraminal stenosis identified on CT. Patients were categorized into the following two groups: those who developed postoperative C5 palsy (P group) and those who did not (NP group). Radiographic parameters compared between the two groups included the preoperative C2-7 angle, C4-5 angle, anteroposterior diameter of the C4-5 intervertebral foramen, C2-7 and C4-5 correction angles, and posterior spinal cord displacement on MRI. The prophylactic effect of foraminotomy was evaluated using Fisher’s exact test. The Mann-Whitney U test was used to compare continuous variables, and multivariate logistic regression analysis was performed to identify risk factors. Recovery time from C5 palsy was also evaluated.

Results

Postoperative C5 palsy occurred in nine patients (12 foramina). Patients who developed postoperative palsy showed significantly greater correction angles at C4-5 and smaller preoperative C4-5 foraminal diameters than those without palsy. No significant difference in the incidence of postoperative C5 palsy was observed between foramina with and without prophylactic foraminotomy. The mean recovery time from C5 palsy was 2.6 months in patients who underwent foraminotomy.

Conclusions

Prophylactic foraminotomy did not reduce the incidence of postoperative C5 palsy following posterior cervical correction surgery using CPS. However, most palsy cases were transient, with a mean recovery period of 2.6 months. The results suggest that a greater correction angle at C4-5 and the presence of preoperative foraminal stenosis are risk factors for C5 palsy in CPS-based corrective surgery. In cases with severe preoperative foraminal stenosis, the magnitude of the correction angle at C4-5 may contribute to the development of C5 palsy, even when prophylactic foraminotomy is performed.

## Introduction

Postoperative C5 nerve palsy is a well-recognized complication of surgery for cervical myelopathy. Many investigators have proposed possible mechanisms underlying this condition, including (1) direct injury to the nerve root, (2) damage to the anterior horn cells, and (3) tethering or impingement of the nerve root within the foramen caused by posterior shift of the spinal cord after posterior decompression. However, the precise mechanism remains unclear.

Sakaura et al. [1] reported an incidence of 4.6% in both anterior decompression and fusion and laminoplasty for cervical spondylotic myelopathy. However, the majority of cases have been widely recognized as occurring after posterior procedures [2-4].

Posterior correction and fixation surgery using cervical pedicle screws (CPS) or lateral mass screws represents a major advancement for patients with cervical kyphotic deformity and/or instability, as it provides sufficient stability to achieve and maintain cervical alignment in addition to allowing extensive posterior decompression [5]. Suda et al. reported that laminoplasty alone in patients with local kyphosis greater than 13° resulted in poorer clinical outcomes, suggesting that posterior kyphosis correction combined with posterior decompression should be considered in such cases [6]. Miyamoto et al. also reported that correction surgery yields superior clinical outcomes compared with laminoplasty alone in patients with local kyphosis [7].

Despite these advantages, several studies have reported that posterior correction surgery is associated with a higher incidence (12-50%) of postoperative C5 nerve palsy compared with conventional laminoplasty [8-11]. This increased incidence may be attributable to kyphosis correction and the development of postoperative iatrogenic foraminal stenosis [8,9]. In a previous study, Nakashima et al. reported that posterior spinal cord shift at the C4-5 level and postoperative C4-5 intervertebral foraminal stenosis were risk factors for C5 palsy [10]. Hojo et al. also reported that excessive kyphosis correction and the presence of preoperative foraminal stenosis at the C4-5 level may be associated with the occurrence of C5 nerve palsy [8]. Kurakawa et al. reported that a preoperative anteroposterior diameter of the C4-5 foramen of less than 4.1 mm on CT was a risk factor for C5 palsy [9].

Therefore, prophylactic foraminotomy may be considered as a preventive strategy. However, it remains unclear whether prophylactic foraminotomy at C4-5 reduces the incidence of postoperative C5 palsy in reconstructive surgery. In our institution, prophylactic foraminotomy at C4-5 has been performed in patients with a preoperative foraminal diameter of less than 4.1 mm. The purpose of this study was to evaluate the effectiveness of prophylactic foraminotomy in preventing postoperative C5 palsy in reconstructive surgery using CPS.

This study was previously presented as an abstract at the 2019 Cervical Spine Research Society Europe Meeting, held on May 22-24, 2019.

## Materials and methods

This retrospective single-center cohort study was approved by the institutional ethics committee of our institution. Between 2013 and 2018, 37 patients underwent cervical fixation surgery for cervical spondylotic myelopathy at Kindai University Hospital. This study enrolled 37 patients with cervical spondylotic myelopathy accompanied by local kyphosis >13° or local segmental instability who underwent fixation or correction surgery using CPS (21 males, 16 females, mean age, 71 years). Patients with cervical ossification of the posterior ligament, ankylosing spine, tumor, infection, and prior surgery were excluded.

Surgical procedures

A total of 11 cases of combined anteroposterior fusion and 26 cases of posterior fusion alone were included. Patients selected for combined anterior and posterior cervical surgery had a C2-7 angle exceeding 13° and a rigid apex of kyphosis. All patients presented with dropped head syndrome. In patients with rigid local kyphosis, anterior release and cage insertion were performed first, followed by posterior decompression and corrective surgery using CPS. In contrast, patients with reducible local kyphosis, mainly associated with anterior vertebral slippage, underwent posterior procedures alone. In all cases, posterior fixation was required to achieve stabilization and correction of the local alignment. The number of fused levels ranged from 1 to 9 (1 level: 2 cases; 2 levels: 4 cases; 3 levels: 13 cases; 4 levels: 7 cases; 5 levels: 5 cases; 6 levels: 5 cases; 9 levels: 1 case). Prophylactic foraminotomy was performed in 15 patients (27 foramina) with a preoperative anteroposterior C4-5 foraminal diameter of ≤4.1 mm on CT.

C5 palsy

Postoperative C5 palsy was defined as deterioration in deltoid muscle strength by at least one grade on the manual muscle test (MMT) after surgery without worsening of myelopathy.

Clinical assessments

The recovery rates based on the Japanese Orthopaedic Association (JOA) score were calculated in both groups. Patients who underwent prophylactic foraminotomy were divided into the following two groups: those who developed postoperative C5 palsy (P group) and those who did not (NP group). The time to neurological recovery was also evaluated.

Radiological assessments

**Figure 1:**
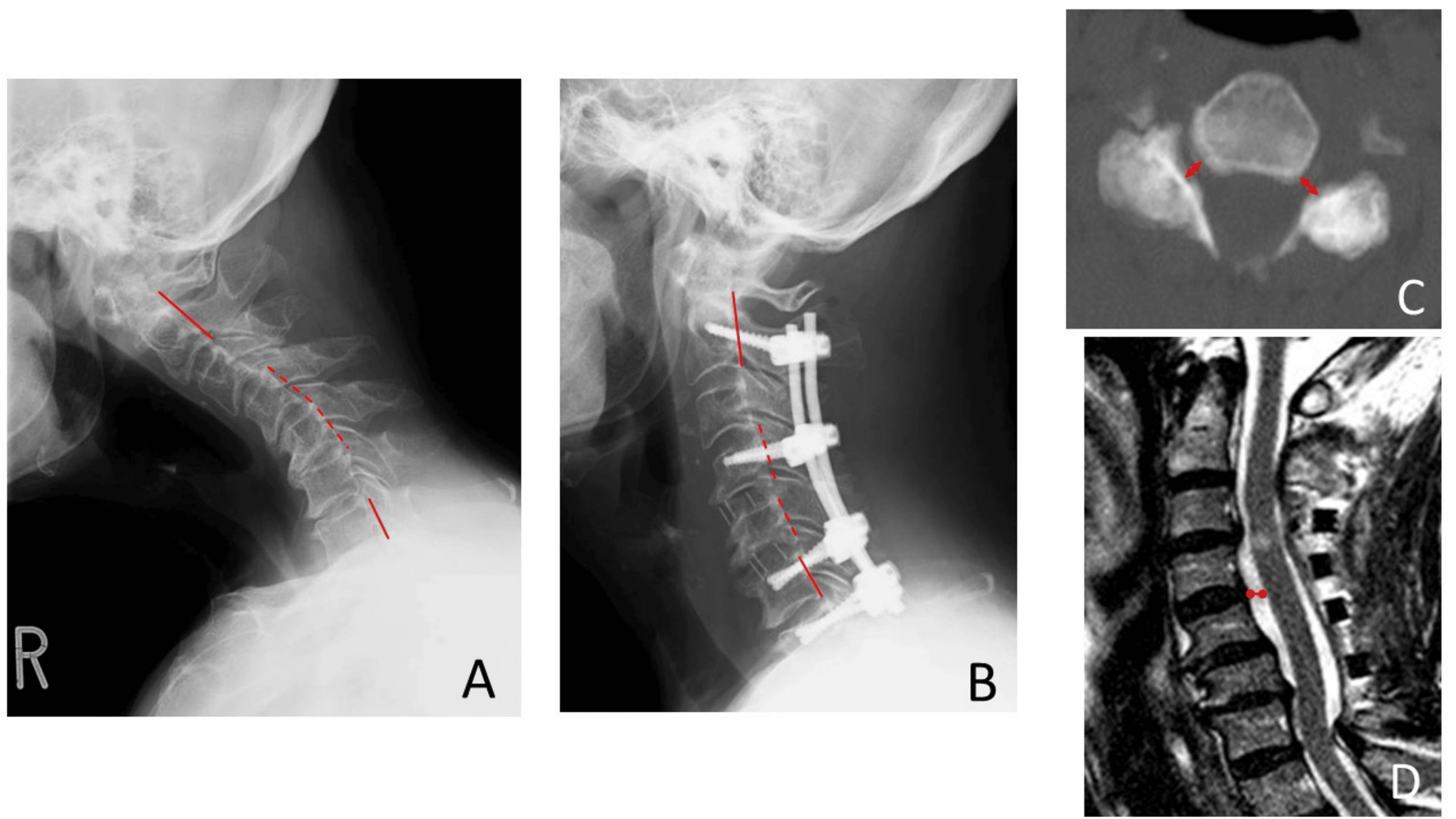
Radiological assessments. (A) Preoperative C2–7 angle (solid line) and C4–5 local angle (dashed line). (B) Postoperative C2–7 angle (solid line) and C4–5 local angle (dashed line). (C) Anteroposterior diameter of the C4–5 intervertebral foramen. (D) Posterior shift of the spinal cord on MRI.

Radiological parameters, including C2-7 and C4/5 angles, were measured pre- and postoperatively. Correction angles for C2-7 and C4/5 were calculated as the difference between post- and preoperative measurements. The preoperative C4/5 foramen diameter was measured from CT axial images. Posterior spinal cord shift at the C4/5 level was assessed using MRI by comparing pre- and postoperative images (Figure 1).

Statistical examination

Statistical analyses were performed using Stat Flex version 7 (Artech, Osaka, Japan). The prophylactic effect of foraminotomy was evaluated using Fisher’s exact test. Radiological and clinical variables were compared between the P and NP groups using the Mann-Whitney U test. Variables that were significant in the univariate analysis were further evaluated using multivariate logistic regression analysis to identify independent factors, including both preoperative and postoperative variables. A p-value <0.05 was considered statistically significant.

## Results

Overall patient demographics and radiographic data for the 74 foramina are summarized in Table 1. Postoperative C5 palsy occurred in nine patients (12 foramina). Of these 12 foramina, 10 had undergone foraminotomy, whereas two had not. Among the 27 foramina that underwent foraminotomy, C5 palsy developed in 10 foramina (P group), while 17 foramina did not develop palsy (NP group). The incidence of C5 palsy was higher in the foraminotomy group (p = 0.0004, Fisher’s exact test), suggesting that foraminotomy did not prevent the occurrence of C5 palsy. However, this finding may reflect selection bias, as foraminotomy was preferentially performed in patients with more severe foraminal stenosis. Among the 27 intervertebral foramina that underwent foraminotomy, five foramina in the NP group had a foraminal diameter exceeding 4.1 mm; nevertheless, foraminotomy was performed. All cases in the P group met the criteria for foraminotomy. In the group without foraminotomy (45 foramina), five foramina demonstrated stenosis (mean anteroposterior diameter: 3.58 mm) but were not treated with foraminotomy, as these cases involved one- or two-level spinal fusion procedures.

**Table 1:** Overall patient demographic, clinical, and radiographic characteristics. AP: anteroposterior fusion; P: posterior fusion only

Number of (patients/foramina)	37/74
Sex (male/female)	21/16
Age (years)	72.1 ± 10.7
Follow-up periods (months)	25.6 ± 9.2
Surgical procedure (AP/P)	11/26
Prophylactic foraminotomy	27
Preoperative C2–7 angle (degrees)	-7.0 ± 17.2
C2–7 correction angle (degrees)	8.4 ± 18.7
Preoperative C4–5 angle (degrees)	-6.5 ± 9.7
C4–5 correction angle (degrees)	5.0 ± 9.7
Preoperative C4–5 foramen diameter (mm)	3.9 ± 1.2

Comparative data between the P and NP groups among cases with foraminotomy are presented in Tables 2 and 3. The preoperative C2-7 angle was -9.9 ± 19.5° in the P group and -8.5 ± 7.9° in the NP group (Table 2). Preoperative alignment did not differ significantly between the groups. The correction of the C2-7 angle was also not significantly different between the P and NP groups (12.5 ± 14.9° vs. 8.2 ± 13.0°, respectively) (Table 2).

**Table 2:** Comparison of C5 palsy and no-palsy groups in patients undergoing foraminotomy. Comparison of patients with and without C5 palsy among the 27 foramina who underwent prophylactic foraminotomy was performed using the Mann–Whitney U test. Data are presented as mean ± standard deviation. A p-value <0.05 was considered statistically significant. P group: C5 palsy; NP group: no C5 palsy

Parameter	P group	NP group	P-value
Number of foramina	10	17	-
Age (years)	74.0 ± 9.1	69.5 ± 10.5	0.313
Preoperative C2–7 angle (°)	-9.9 ± 19.5	-8.5 ± 7.9	0.959
Postoperative C2–7 angle (°)	2.6 ± 8.6	-0.35 ± 7.3	0.300
C2–7 correction angle (°)	12.5 ± 14.9	8.2 ± 13.0	0.544

The preoperative C4-5 local angle was -10.7 ± 9.8° in the P group and -4.8 ± 10.3° in the NP group (Table 3), with no significant difference between the groups. However, the correction angle at C4-5 was significantly greater in the P group than in the NP group (13.3 ± 9.5° vs. 5.8 ± 8.7°, p = 0.041, Mann-Whitney U test) (Table 3). The preoperative C4-5 foraminal diameter was significantly smaller in the P group than in the NP group (3.0 ± 0.7 mm vs. 4.1 ± 1.4 mm, p = 0.048, Mann-Whitney U test) (Table 3). The posterior shift of the spinal cord at the C4-5 level did not differ significantly between the P and NP groups (1.7 ± 1.2 mm vs. 2.1 ± 0.9 mm) (Table 3).

**Table 3:** C4–5 foraminal parameters in patients undergoing foraminotomy: comparison between patients with and without C5 palsy. Comparison of patients with and without C5 palsy among those undergoing foraminotomy. Data are presented as mean ± standard deviation. A p-value <0.05 was considered statistically significant. P group: C5 palsy; NP group: no C5 palsy

Parameter	P group	NP group	P-value
Numbers of foramen	10	17	-
Preoperative C4–5 angle (°)	-10.7 ± 9.8	-4.8 ± 10.3	0.179
Postoperative C4–5 angle (°)	1.1 ± 4.9	1.7 ± 6.1	0.648
Preoperative C4–5 foramen diameter (mm)	3.0 ± 0.7	4.1 ± 1.4	0.048
C4–5 correction angle (°)	13.3 ± 9.5	5.8 ± 8.8	0.041
Posterior spinal cord shift (mm)	1.7 ± 1.2	2.1 ± 0.9	0.243

All 74 foramina were further analyzed by comparing 12 foramina in the P group (10 with foraminotomy and two without) and 62 foramina in the NP group (17 with foraminotomy and 45 without), irrespective of foraminotomy status (Table 4). No significant differences in preoperative cervical alignment were observed between the two groups, including the preoperative C2-7 angle (-8.4 ± 18.3° vs. -6.7 ± 17.1°) and preoperative C4-5 angle (-8.3 ± 10.2° vs. -6.0 ± 9.5°). Similarly, correction of the C2-7 angle did not differ significantly between groups (12.5 ± 14.5° vs. 7.4 ± 19.5°). However, the correction angle at C4-5 was significantly greater in the P group than in the NP group (13.3 ± 10.0° vs. 5.9 ± 9.0°, p = 0.045) (Table 4). In addition, the preoperative C4-5 foraminal diameter was significantly smaller in the P group than in the NP group (3.1 ± 0.7 mm vs. 4.1 ± 1.2 mm, p = 0.028) (Table 4). These differences were statistically significant between the two groups. Posterior shift of the spinal cord did not differ significantly between the groups (1.8 ± 1.2 mm vs. 1.9 ± 0.9 mm).

**Table 4:** Patient demographic, clinical, and radiographic data in 74 foraminal with and without postoperative C5 palsy. Data were compared using the Mann–Whitney U test. A p-value <0.05 was considered statistically significant. P: palsy; NP: non-palsy

Parameter	P group (n = 12 foramina)	NP group (n = 62 foramina)	P-value
Foraminotomy status	10 with/2 without	17 with/45 without	-
Preoperative C2–7 angle (°)	-8.4 ± 18.3	-6.7 ± 17.1	0.735
Postoperative C2–7 angle (°)	3.1 ± 7.5	0.7 ± 8.0	0.170
C2–7 correction angle (°)	12.5 ± 14.5	7.4 ± 19.5	0.211
Preoperative C4–5 angle (°)	-8.3 ± 10.2	-6.0 ± 9.5	0.517
Postoperative C4–5 angle (°)	1.8 ± 4.9	1.1 ± 6.2	0.555
C4–5 correction angle (°)	13.3 ± 10.0	5.9 ± 9.0	0.045
Preoperative C4–5 foramen diameter (mm)	3.1 ± 0.7	4.1 ± 1.2	0.028
Posterior spinal cord shift (mm)	1.8 ± 1.2	1.9 ± 0.9	0.253

We investigated factors associated with C5 palsy based on preoperative data using multivariate logistic regression analysis. The analysis included the preoperative C2-7 angle, C4-5 angle, and C4-5 anteroposterior diameter as candidate risk factors. Among these variables, the anteroposterior diameter was identified as a significant factor associated with C5 palsy. The model demonstrated an Akaike information criterion (AIC) of 65.778 and an area under the curve AUC of 0.737 (Table 5). Next, we examined factors associated with C5 palsy based on postoperative data using multivariate logistic regression analysis. The analysis included the C2-7 correction angle, C4-5 correction angle, and posterior shift of the spinal cord as candidate risk factors. Among these variables, the C4-5 correction angle was identified as a significant factor associated with C5 palsy. The model demonstrated an AIC of 66.236 and an AUC of 0.693 (Table 6).

**Table 5:** Preoperative risk factors associated with C5 palsy. Multivariate logistic regression analysis was performed using the preoperative C2–7 angle, C4–5 angle, and C4–5 AP diameter as candidate risk factors. Among these variables, the AP diameter was identified as a significant factor associated with C5 palsy. *: A p-value <0.05 indicates statistical significance. AIC = 65.778, AUC = 0.737. AP: anteroposterior; AUC: area under the curve; AIC: Akaike information criterion; OR: odds ratio; CI: confidence interval

Parameter	P-value	OR	95% CI
C2–7 angle (°)	0.433	1.025	0.963–1.091
C4–5 angle (°)	0.115	0.927	0.844–1.019
AP diameter (mm)	0.015*	0.439	0.226–0.853

**Table 6:** Postoperative risk factors associated with C5 palsy. Multivariate logistic regression analysis was performed using the preoperative C2–7 correction angle, C4–5 correction angle, and posterior shift of the spinal cord as candidate risk factors. *: A p-value <0.05 indicates statistical significance. AIC = 66.236, AUC = 0.693. AUC: area under the curve; AIC: Akaike information criterion; OR: odds ratio; CI: confidence interval

Parameter	P-value	OR	95% CI
C2–7 correction angle (degrees)	0.462	0.984	0.941–1.028
C4–5 correction angle (degrees)	0.029*	1.112	1.011–1.224
Posterior shift of the spinal cord (mm)	0.596	1.192	0.623–2.281

Finally, a multivariate logistic regression analysis incorporating both preoperative and postoperative variables was performed using a stepwise forward selection method. In the final model, the C4-5 correction angle, preoperative anteroposterior diameter, and C2-7 correction angle were retained. Among these variables, the C4-5 correction angle (p = 0.014) and preoperative anteroposterior diameter (p = 0.024) were identified as significant factors associated with C5 palsy, whereas the C2-7 correction angle was not statistically significant (p = 0.144). The model demonstrated acceptable discrimination (AUC = 0.808) and a good fit (AIC = 60.440) (Table 7).

**Table 7:** Multivariable analysis of pre and postoperative risk factors associated with C5 palsy. The C4–5 correction angle and the preoperative AP diameter were identified as significant factors associated with C5 palsy. The model demonstrated acceptable discrimination (AUC = 0.808) with a good fit (AIC = 60.440). *: A p-value <0.05 indicates statistical significance. AIC = 60.440, AUC = 0.808 AP: anteroposterior; AUC: area under the curve; AIC: Akaike information criterion; OR: odds ratio; CI: confidence interval

Parameter	P-value	OR	95% CI
C4–5 correction angle (degrees)	0.014*	1.564	1.097–2.230
AP diameter (mm)	0.024*	0.681	0.488–0.950
C2–7 correction angle (degrees)	0.144	1.328	0.908–1.943

We investigated the prognosis of C5 palsy. Muscle weakness in the deltoid and biceps muscles was observed in all cases between postoperative day one and day eight, with muscle strength ranging from MMT grade 2 to 3. Complete recovery (MMT grade 5) was achieved in 11 of 12 cases (91.7%), while one case (8.3%) showed incomplete recovery. The mean recovery period was 2.6 months. The time to complete recovery was approximately 2.1 months in the foraminotomy group and 3.5 months in the non-foraminotomy group; however, this difference was not statistically significant. Overall, muscle strength improved from MMT grade 2-3 in the early postoperative period to grade 5 in most cases, with complete recovery observed in both the deltoid and biceps muscles. One patient in the foraminotomy group showed only partial recovery, improving from MMT grade 2 to grade 3 at six months postoperatively without achieving full recovery. In this case, the biceps muscle recovered completely to MMT grade 5, whereas the deltoid muscle remained at MMT grade 3. No reoperation was required during the follow-up period. The recovery rate of the JOA score was 45 % in the P group and 48% in the NP group, with no significant difference between the groups.

## Discussion

Previous studies have reported that posterior reconstruction using screw-rod systems, such as CPS, is associated with a higher incidence of postoperative C5 palsy (12-50%) compared with conventional laminoplasty. Several reports have suggested that prophylactic foraminotomy in conventional laminoplasty may help prevent C5 nerve palsy [12,13]. This procedure is based on the concept of relieving nerve root impingement within the vertebral foramen when the spinal cord shifts posteriorly following decompression. We believe that C5 palsy following posterior fixation arises from causes other than nerve root traction resulting from posterior spinal cord shift. Therefore, we examined the pre- and postoperative status of the C4-5 foramen. In the present study, C5 palsy developed in 12 of 74 foramina. Consistent with previous reports, our findings suggest that greater local kyphosis correction and preoperative foraminal stenosis are associated with an increased risk of C5 palsy in posterior corrective surgery using CPS [8-10]. The onset of C5 palsy occurred several days postoperatively, which is also consistent with previous reports [8-11]. However, the effectiveness of prophylactic foraminotomy in this specific surgical setting remains unclear. The incidence of C5 palsy was significantly higher in the foraminotomy group (p = 0.0004, Fisher’s exact test). This result likely reflects selection bias, as foraminotomy was preferentially performed in patients with more severe foraminal stenosis. In fact, most cases undergoing foraminotomy demonstrated a narrow preoperative C4-5 foraminal diameter(Tables 3, 4). Our findings indicate that a smaller preoperative anteroposterior diameter of the C4-5 intervertebral foramen is associated with an increased risk of postoperative C5 palsy (Table 5). Despite this, prophylactic foraminotomy did not appear to reduce the incidence of C5 palsy. One possible explanation is that substantial local correction at the C4-5 level may offset the potential benefit of foraminotomy. In the present study, multivariate logistic regression analysis identified a greater C4-5 correction angle as an independent factor associated with C5 palsy (Tables 6, 7). In contrast, posterior shift of the spinal cord, a surrogate marker of nerve root traction, was not significantly associated with C5 palsy. These findings suggest that factors related to foraminal morphology, rather than nerve root traction, may play a more important role (Table 5).

Hojo et al. reported postoperative C5 palsy in 6 of 20 patients (30%) undergoing cervical kyphosis correction surgery, despite prophylactic C4-5 foraminotomy being performed in three of these six patients [8]. Secondary C4-5 foraminotomy was subsequently performed in the remaining patients, and the mean recovery time was 3.5 months. Similarly, Nakashima et al. reported postoperative C5 palsy in 10 of 84 patients (12%) undergoing posterior corrective surgery, including cases with cervical ossification of the posterior longitudinal ligament [10]. Among these 10 patients, only one underwent prophylactic C4-5 foraminotomy, while four subsequently required additional C4-5 foraminotomy. Although eight patients achieved full recovery, two did not. Among those who achieved full recovery, the recovery period exceeded 9.7 months. In the present study, most cases of C5 palsy were transient, with a mean recovery period of 2.6 months. Although the recovery time tended to be shorter in the foraminotomy group than in the non-foraminotomy group, this difference was not statistically significant. Therefore, the potential effect of foraminotomy on recovery should be interpreted with caution. In the present study, most cases of C5 palsy were transient, with a mean recovery period of 2.6 months. Although the recovery time tended to be shorter in the foraminotomy group than in the non-foraminotomy group, this difference was not statistically significant. Therefore, the potential effect of foraminotomy on recovery should be interpreted with caution.

This study has several limitations, including its retrospective design, small sample size, and potential selection bias. In addition, we were unable to directly evaluate postoperative changes in foraminal morphology. Further studies are needed to clarify the role of foraminotomy in preventing and influencing the course of C5 palsy in CPS-based posterior corrective surgery. Therefore, it may be important to consider not only the presence of foraminal stenosis but also the magnitude of local correction at C4-5 when planning surgery.

## Conclusions

Prophylactic foraminotomy did not appear to reduce the incidence of postoperative C5 palsy following posterior cervical corrective surgery using CPS. However, most cases of C5 palsy were transient, with a mean recovery period of 2.6 months. Greater kyphosis correction at the C4-5 level and preoperative foraminal stenosis were identified as significant factors associated with C5 palsy. These findings highlight the importance of careful preoperative radiographic assessment and appropriate surgical planning in patients undergoing cervical kyphosis correction surgery.
